# Improved Speech Recognition with Automated ForwardFocus in Cochlear Kanso 3 Sound Processor Users

**DOI:** 10.1097/ONO.0000000000000087

**Published:** 2026-03-18

**Authors:** Marian Jones, Wei Hong, I. Manjula Schou, Marjan (Emjay) Mashal

**Affiliations:** 1 Cochlear Limited, 1 University Avenue, Macquarie University, NSW, Australia

**Keywords:** Cochlear implant, ForwardFocus, Noise reduction technology, Off-the-ear, Sound processor, Speech recognition

## Abstract

**Objective:**

The primary objective of this study was to evaluate adult cochlear implant (CI) users’ speech perception in spatially separated noise (S0Nrearhalf) with SCAN 2 FF (automated ForwardFocus) versus SCAN 2 (ForwardFocus off) with the Cochlear Kanso 3 Sound Processor. Secondary objectives included comparing speech perception in the S0N3 configuration, speech perception in quiet between the Kanso 3 Sound Processor and the Kanso 2 Sound Processor, and evaluating user satisfaction and subjective hearing performance with the Kanso 3 Sound Processor versus their own sound processor.

**Study Design:**

Single-center, within-subject, interventional study (NCT05619575).

**Setting:**

Purpose-built clinical research unit for auditory device evaluation.

**Patients:**

Twenty adults who were postlingually deafened, with at least 6 months of experience with a CI and 3 months of experience with the SCAN program, were recruited.

**Intervention:**

The Kanso 3 Sound Processor with the SCAN 2 FF program.

**Main Outcome Measure:**

Paired difference in speech reception threshold (dB SNR) between the SCAN 2 FF and SCAN 2 programs with the Kanso 3 Sound Processor, as measured using the Australian Sentence Test in Noise.

**Results:**

Mean speech recognition thresholds were significantly lower (better) with SCAN 2 FF compared with SCAN 2 in both the S0Nrearhalf and S0N3 configurations by 2.00 dB and 1.43 dB, respectively (*P* < 0.001). Speech perception in quiet was comparable between the Kanso 3 and Kanso 2 Sound Processors. Participants reported high satisfaction with the Kanso 3 Sound Processor, and subjective hearing performance was similar to their own sound processor.

**Conclusions:**

This study reinforces the effectiveness of SCAN 2 FF in the Kanso 3 Sound Processor for improving speech perception in noise.

## INTRODUCTION

Cochlear implants (CIs) have significantly transformed the hearing experience for individuals with severe to profound hearing loss (1). These implants include an external sound processor, available in behind-the-ear (BTE) or off-the-ear (OTE) configurations. OTE sound processors have provided CI users the flexibility to align their hearing experience with personal, lifestyle, and aesthetic preferences.

While CI users generally display good speech understanding in quiet conditions (2), their auditory performance significantly deteriorates in the presence of background noise (2,3). Additionally, CI users exert significantly more listening effort in challenging auditory settings compared with individuals with normal hearing, leading to increased listening-related fatigue (4–6). Consequently, the development of noise reduction algorithms to enhance CI performance in noisy environments remains a critical area of research (7–9).

### Kanso Sound Processor

The Kanso Sound Processor was the first OTE device developed by Cochlear Ltd (Sydney, Australia) and was designed for discretion and simplicity, while still maintaining adequate hearing performance outcomes. To address the ongoing challenge of listening in noisy environments, the Kanso Sound Processor incorporated several sound processing technologies (Table 1). Dual omnidirectional microphones, including standard (moderately directional), zoom (fixed directional), and Beam (adaptive directional), worked together to minimize background noise from behind and the sides of the listener. The automatic scene classifier, SCAN, was also included with the Kanso Sound Processor, which analyses various sound features to automatically classify the listening environment and select the most suitable sound processing enhancement, such as microphone directionality, based on the environment classification (10,11). In a study of 20 adult CI users, the Kanso Sound Processor’s SCAN feature demonstrated improved performance in spatially separated noise compared with single-microphone and standard programs (11). Participants also rated the Kanso Sound Processor significantly better than their BTE sound processors in terms of comfort (*P* < 0.001), look and feel (*P* < 0.001), ease of use (*P* < 0.01), music quality (*P* < 0.05), and overall hearing performance (*P* < 0.05) (11).

**TABLE 1.** Sound processing technologies introduced in each generation of Cochlear OTE Sound Processors

Feature	Function	OTE sound processor^a^
Dual omnidirectional microphones (standard, zoom, Beam)	Two-microphone design that work together to capture sounds more effectively than a single-microphone. There are 3 microphone directionalities: standard (moderately directional), zoom (fixed directional), and Beam (adaptive directional).	Kanso Sound Processor
SCAN	Automatically classifies the listener’s environment into 1 of 6 scenes (speech in noise, noise, speech, quiet, wind, music).	Kanso Sound Processor
Signal-to-noise ratio-based noise reduction	Reduces the interference of background noise.	Kanso Sound Processor
Adaptive dynamic range optimization	Ensures that sounds are comfortable for the listener.	Kanso Sound Processor
Automatic sensitivity control	Enhances the audibility of soft speech sounds.	Kanso Sound Processor
Wind noise reduction	Detects wind and adjusts settings accordingly to keep the listener comfortable.	Kanso Sound Processor
User-controlled ForwardFocus	Attenuates multiple noise sources from behind the listener. Users manually switch this feature on or off via the Cochlear Nucleus Smart App.	Kanso 2 Sound Processor
Automated ForwardFocus (with SCAN 2 FF)	Attenuates multiple noise sources from behind the listener. Users have the option to use automated ForwardFocus, which automatically adjusts the strength of ForwardFocus based on the detected sound class.	Kanso 3 Sound Processor
Bluetooth LE Audio and Auracast broadcast audio	Enables connection to other Bluetooth LE Audio compatible devices (once the technology is available).	Kanso 3 Sound Processor

a The OTE sound processor in which this feature was first introduced.

### Kanso 2 Sound Processor

**FIG. 1.**
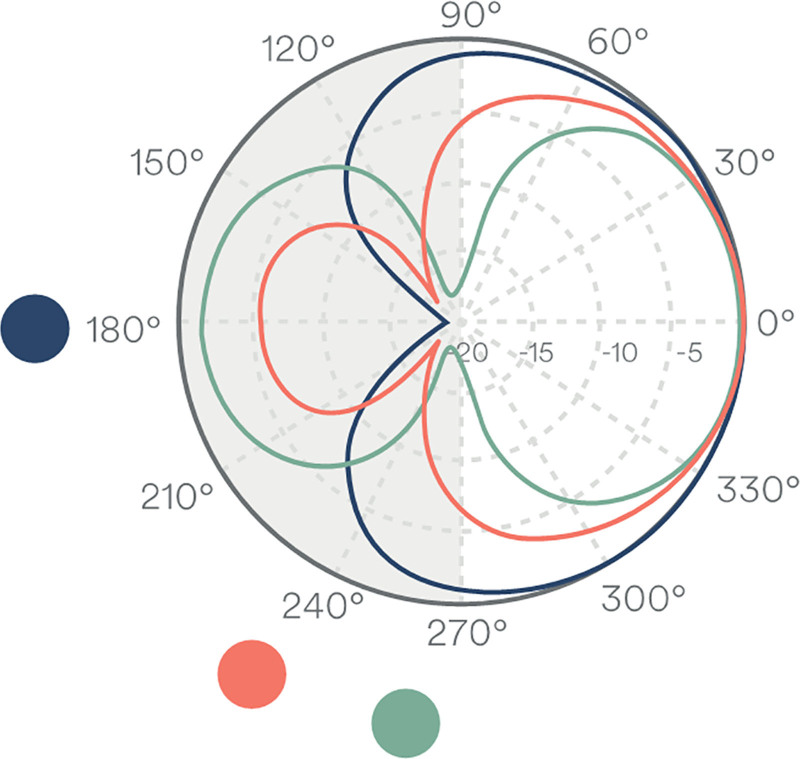
Schematic polar plot illustrating combined use of ForwardFocus and microphone directionality (Beam) in an environment with multiple noise sources. Shaded regions indicate areas of attenuation behind the listener when ForwardFocus is active. Coloured dots represent individual noise sources, and the corresponding coloured polar plots depict the directional response of Beam. This figure has been reproduced from: 1) Clinical white paper. Improving listening in noise with ForwardFocus technology in Nucleus Sound Processors. Cochlear Limited. April 2025, and 2) Nel et al (22) (under the terms of the CC BY-NC-ND license).

A user-controlled noise reduction technology called ForwardFocus (FF) was included with the Kanso 2 Sound Processor (Table 1). Unlike Beam, which adaptively attenuates noise from a single dominant source, FF can reduce noise from multiple sources located behind the listener, while passing sounds from the front (Figure 1). This is achieved using inputs from 2 omnidirectional microphones to create a target and noise spatial pattern. The algorithm compares these spatial patterns to attenuate noise from the signal. In a study involving 22 adult CI users, FF implemented in the Kanso 2 Sound Processor led to overall improvements in hearing performance in noise (S0N3 configuration) compared with FF off (8). Similarly, in a separate study of 20 adult CI users evaluating the application of 3 sound processing technologies (standard, Beam, and FF) in the Kanso and Kanso 2 Sound Processors, FF combined with the Kanso 2 Sound Processor demonstrated the highest speech understanding and lowest subjective listening effort (12).

### Kanso 3 Sound Processor

The Kanso 3 Sound Processor now offers optional automation of FF (SCAN 2 FF) within the updated SCAN 2 program. When enabled, SCAN 2 FF automatically adjusts FF strength based on the detected sound class, applying maximal strength in speech-in-noise and in general noise, and minimal strength in quiet or when speech or music is present. This automated function eliminates the need for CI users to manually select environment-specific listening programs. Previous research has shown that when given the option to manually switch between 2 programs (“quiet” and “noise”) based on the listening environment, CI users switched programs less than once per day on average. Despite extensive counseling, participants were unable to correctly classify all listening environments, with the sound processor being in the intended program only 60% of the time (13). Similarly, previous research found limited use of user-controlled FF in the Nucleus 7 Sound Processor and Kanso 2 Sound Processor (14). Implementing SCAN 2 FF may therefore enhance convenience and listening outcomes by removing the need for manual adjustments in noisy settings. SCAN 2 FF is also available in the Cochlear Nucleus 8 Sound Processor and has shown to improve speech recognition, relative to SCAN 2 (with FF off), in 2 spatially separated speech and noise conditions in a study involving 20 CI users (15). Additionally, CI users reported positive feedback following real-world use of the SCAN 2 FF program in the Nucleus 8 Sound Processor (15). The present study aimed to evaluate the effectiveness of SCAN 2 FF in an OTE configuration.

**FIG. 2.**
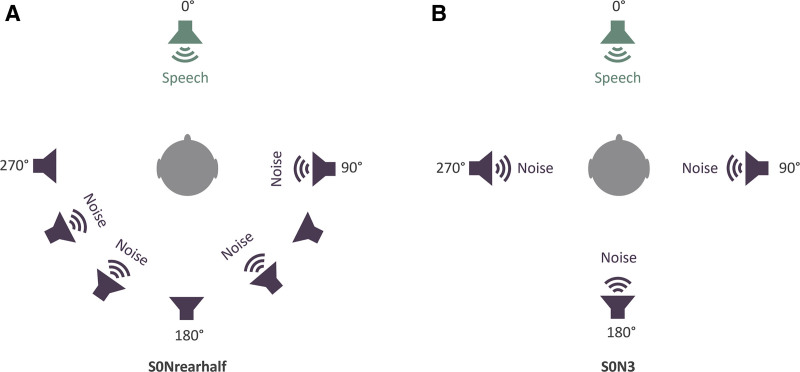
The loudspeaker configurations utilized for speech in noise testing. *A,* In S0Nrearhalf, 4 independent interfering talkers were randomly chosen from 7 loudspeakers between 90° and 270° in the rear half. *B,* In S0N3, babble noise was presented from 3 loudspeakers positioned at 90°, 180°, and 270°.

The aim of the present study was to determine the performance and acceptance of the Kanso 3 Sound Processor, with a particular focus on the SCAN 2 FF program. The primary objective of the study was to compare speech perception in spatially separated noise with SCAN 2 FF (automated FF) compared with SCAN 2 (FF off) in the Kanso 3 Sound Processor in the S0Nrearhalf loudspeaker configuration (Fig. 2). The secondary objectives included: 1) comparing speech perception in spatially separated noise between the SCAN 2 FF and SCAN 2 programs in the S0N3 loudspeaker configuration (Fig. 2), 2) comparing speech perception in quiet between Kanso 3 and Kanso 2 Sound Processors, 3) assessing the subjective acceptance and satisfaction of participants, and 4) evaluating patient-reported hearing ability with the Kanso 3 Sound Processor compared with their own sound processor.

## METHODS

This study was a 12-month, prospective, single-center, within-subject, interventional investigation. Ethics approval was obtained from the Bellberry Human Research Ethics Committee (2022-10-1124). The study is registered publicly on ClinicalTrials.gov (NCT05619575).

### Participants

Twenty adults with postlingual onset of severe to profound hearing loss, aged 18 years or older, who were implanted with the CI600 Series, CI500 Series, Freedom Series, or N24 Series, were enrolled. Inclusion criteria included at least 6 months of experience with a CI and 3 months of experience with any Nucleus Sound Processor and a SCAN program. Participants were also required to score 30% or more at + 15 signal-to-noise ratio (SNR) with a CI alone on a sentence in the babble test to be eligible to participate. All participants signed an informed consent form before participating in the study. Exclusion criteria included additional disabilities preventing participation in evaluations, unrealistic expectations regarding the procedures’ benefits, risks, and limitations, inability or unwillingness to comply with study requirements, and participation in another interventional study within 30 days before enrollment. Additionally, investigator site personnel directly affiliated with the study and Cochlear employees were excluded from participation.

### Speech Perception Testing

For speech testing, all participants were tested unilaterally. For unilateral participants, the contralateral (nontest) ear was blocked with an earplug across all test conditions. For bilateral participants, the test ear was either their preferred listening ear or, if no preference was indicated, the first implanted ear.

#### Speech in Noise

Speech-in-noise testing was performed using the Australian Sentence Test in Noise (AuSTIN) (16), in which Bamford-Kowal-Bench-like (17) target sentences were presented in adaptive noise to obtain the SNR for 50% speech intelligibility. The participant was positioned so that the midpoint of the 2 ears was centered at the reference point of the sound field. The target sentence was presented from the front (0 degrees) and was fixed at 65 decibel sound pressure level (dB SPL). The test was conducted in 2 spatially separated test conditions: S0Nrearhalf and S0N3 (Fig. 2), as previously reported (8). In the S0Nrearhalf loudspeaker configuration, roving babble noise was presented from 4 loudspeakers in the rear hemisphere (4 talkers in total). These 4 independent interfering talkers were randomly chosen from 7 loudspeakers between 90° and 270° in the rear hemi-field. In the S0N3 loudspeaker configuration, babble noise was presented from 90°, 180°, and 270° (3 talkers in total).

The room adhered to ANSI S3.1 and ISO 8253-1 standards for audiometry. All test runs used 20 sentences. The sound processing settings used for each condition are shown in Table 2. SNR-based noise reduction (SNR-NR) was enabled for all participants during in-booth speech perception testing to reflect default clinical settings and to maintain methodological consistency with a previous study that examined SCAN 2 FF in a BTE sound processor (15). Whisper, WNR, ADRO, and ASC were activated according to participant preferences. This approach ensured that each participant used their optimal sound processor settings, allowing any observed changes in performance to be attributed specifically to the SCAN 2 FF or SCAN 2 programs, rather than to deviations from their usual hearing configurations. FF is designed to function independently of other sound processing technologies such as WNR, ADRO, and ASC. Provided that these settings remain consistent across both SCAN 2 and SCAN 2 FF programs in each participant, any observed performance differences can be attributed to the FF algorithm.

**TABLE 2.** Sound processing settings used during speech in noise testing

Condition	SNR-NR	SCAN Program	ForwardFocus
Kanso 3 SCAN 2 FF (Automated FF ON)	✓	SCAN 2	ON
Kanso 3 SCAN 2 (FF OFF)	✓	SCAN 2	OFF

FF, ForwardFocus; SNR-NR indicates Signal-to-noise ratio-based noise reduction.

#### Speech in Quiet

Speech in quiet testing was performed using consonant-nucleus-consonant (CNC) monosyllabic words (18) at 50 dB SPL from the front. This level was chosen as 50 dB SPL represents soft conversational speech. Testing at this lower level helps reduce ceiling effects that can occur at higher presentation levels, allowing performance to be assessed under more challenging listening conditions and providing a more sensitive measure of variability between sound processors. Two lists were used per condition (Kanso 2 and Kanso 3 Sound Processors), and the average across the lists was analyzed. The percentage of words correct was compared for each condition. The sound processing settings used for each condition are shown in Table 3. SNR-NR was enabled for all participants, and Whisper, WNR, ADRO, and ASC were activated according to participant preferences.

**TABLE 3.** Sound processing settings used during speech in quiet testing

Condition	SNR-NR	SCAN Program	ForwardFocus
Kanso 3	✓	SCAN 2	OFF
Kanso 2	✓	SCAN	OFF

SNR-NR indicates Signal-to-noise ratio-based noise reduction.

### Subjective Evaluations

A shortened form of the 49-questionnaire, Speech, Spatial and Qualities of Hearing scale (SSQ), the SSQ12, was used in this study (19). Participants were asked to rate their ability to hear speech in a variety of competing contexts as well as on their spatial hearing abilities, ease of listening, and identifiability of different loudspeakers. Responses were marked on a scale of 0–10, with higher scores signifying subjectively better performance of the sound processor. This questionnaire was completed at baseline for participants’ own sound processor and after take-home use with the Kanso 3 Sound Processor.

The Baseline Questionnaire (Supplemental Material 1 https://links.lww.com/ONO/A44) gathered information on the participants’ preferred sound processor strategies and usage characteristics with their own sound processor. These questions were related to SCAN and user-controlled FF on the participant’s own device. Following a minimum 2-week take-home period, participants completed the Kanso 3 Questionnaire (Supplemental Material 2 https://links.lww.com/ONO/A45) to evaluate their experience with the Kanso 3 Sound Processor, focusing specifically on their satisfaction with the SCAN 2 FF program and the SCAN 2 program with user-controlled FF.

### Study Schedule

**FIG. 3.**
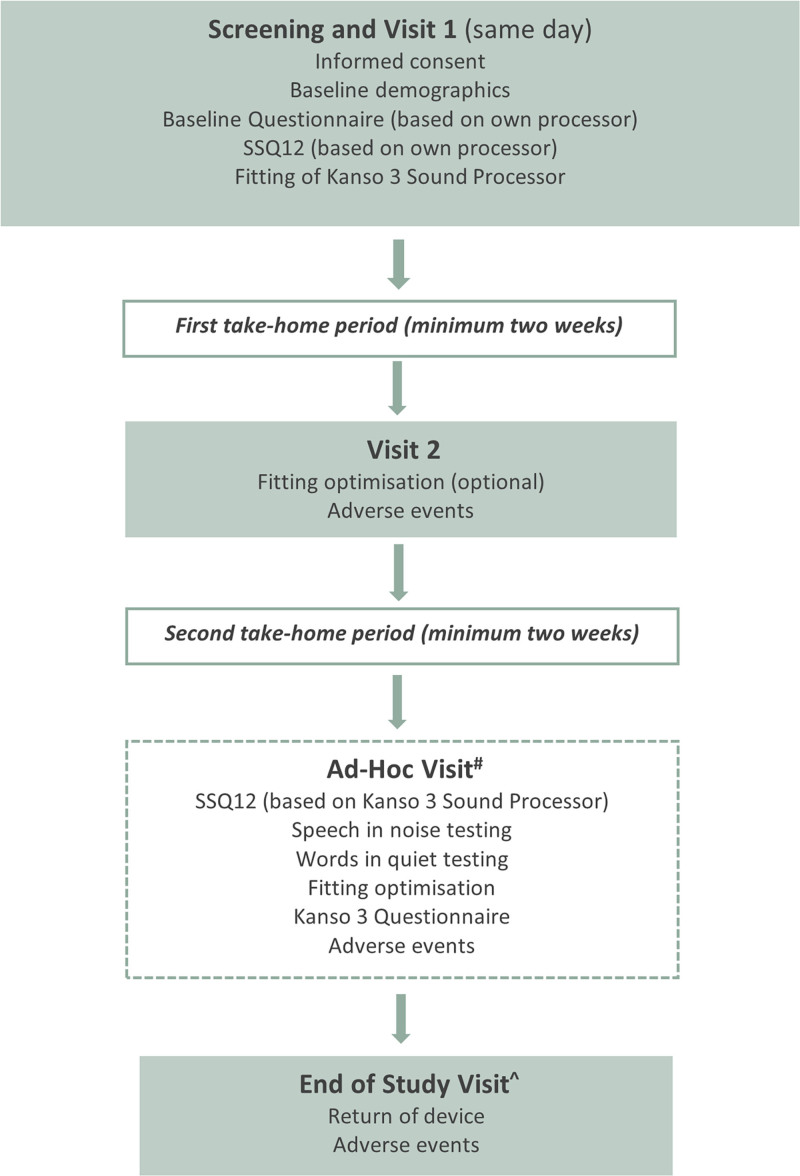
Schedule of enrollment, interventions, and measurements to be collected. ^#^Procedures from Visit 2 and ad hoc visit may be conducted on the same day. ^^^End of Study Visit can occur on the same day as an ad hoc visit.

Participants completed hearing assessments during 2 study visits and ad-hoc visits (Fig. 3). To enable actual use in the home environment, participants took home the Kanso 3 Sound Processor for a minimum of 2 weeks to use as their full-time device. All participants were provided with SCAN 2 FF as Program 1, and SCAN 2 with user-controlled FF as Program 2. During the take-home periods, participants were encouraged to use both programs in different listening situations but were encouraged to use Program 1 (SCAN 2 FF) as much as possible, with the option to switch to Program 2 (SCAN 2 with user-controlled FF), if desired. Participants had access to user-controlled FF in the Nucleus Smart app, allowing them to manually activate FF while using the SCAN 2 program.

### Statistical Analysis

This study aimed to evaluate whether SCAN 2 FF is noninferior to SCAN 2 in the Kanso 3 Sound Processor on speech reception threshold (SRT) in noise scores. A sample size of 17 provided 80% power at a one-sided 2.5% significance level (equivalent to a one-sided 97.5% confidence interval [CI]) to demonstrate noninferiority of SRT sentences in noise scores with SCAN 2 FF compared with SCAN 2, using a noninferiority margin of 1 dB (based on clinical consensus) and a standard deviation of 1.36 dB for the difference in scores. Twenty participants were recruited to allow for any premature participant withdrawal.

Assessment of noninferiority of SCAN 2 FF compared with SCAN 2 with respect to SRT in noise scores, CNC words in quiet scores, and SSQ12 scores was based on a 2-sided 95% CI for the mean paired difference. If the upper limit of the 95% CI for the mean paired difference was below 1 dB (for SRT sentences in noise scores), or above −10% (for CNC words in quiet scores), or above −1 (for SSQ12 scores), SCAN 2 FF was regarded as noninferior to SCAN 2. Once noninferiority was established, testing proceeded to a test of superiority (paired *t* test). No adjustment for multiplicity of testing was made for the secondary hypotheses (CNC words in quiet scores and SSQ12 scores). Data from subjective evaluation questionnaires were summarized descriptively. All analyses were performed using R version 4.1.1.

## RESULTS

### Demographics and Baseline Characteristics

Three of the 20 study participants were withdrawn after screening due to technical issues and personal reasons. The remaining 17 participants received all procedures and subjective evaluations. The mean age of the 20 participants was 63.4 years, with a range of 35–84 years. The mean duration of hearing loss before cochlear implantation in the test ear was 34 years, with a range of 1–61 years. The mean duration of implantation in the test ear was 9.1 years with a range of 0–22 years (Table 4). At baseline, 3/20 participants used an OTE processor (Kanso Sound Processor or Kanso 2 Sound Processor) while 17/20 participants used a BTE processor (Nucleus 7 Sound Processor).

**TABLE 4.** Participant demographics

	Test ear	Contralateral ear
Age at onset of hearing loss (years)		
N	20	20
Mean (SD)	20.2 (16.8)	22.6 (19.2)
Type of hearing loss		
N	20	20
Sensorineural	20 (100.0%)	20 (100.0%)
History of hearing loss		
N	20	20
Congenital with progression	2 (10.0%)	2 (10.0%)
Congenital without progression	0 (0.0%)	1 (5.0%)
Progressive	14 (70.0%)	15 (75.0%)
Progressive with sudden^a^	1 (5.0%)	0 (0.0%)
Sudden	3 (15.0%)	2 (10.0%)
Primary cause of hearing loss		
N	20	20
Autoimmune (etiology unknown)	1 (5.0%)	1 (5.0%)
Genetic	5 (25.0%)	5 (25.0%)
Meningitis	1 (5.0%)	1 (5.0%)
Ototoxic drugs	1 (5.0%)	1 (5.0%)
Unknown	12 (60.0%)	12 (60.0%)
Age at implantation, years^b^		
N	20	11
Mean (SD)	54.2 (14.4)	48.2 (17.0)
Duration of hearing loss before implantation, years^b^		
N	20	11
Mean (SD)	34.0 (18.7)	31.1 (19.3)
Duration of implantation, years^b^		
N	20	11
Mean (SD)	9.1 (5.4)	10.0 (5.3)

a Progressive with sudden refers to where a person experiences a gradual worsening of hearing over time, followed by episodes of rapid and significant hearing loss occurring over hours or days.

b Sample size in the contralateral ear was < 20 as not all participants’ contralateral ear was implanted.

### Speech Recognition in Noise

**FIG. 4.**
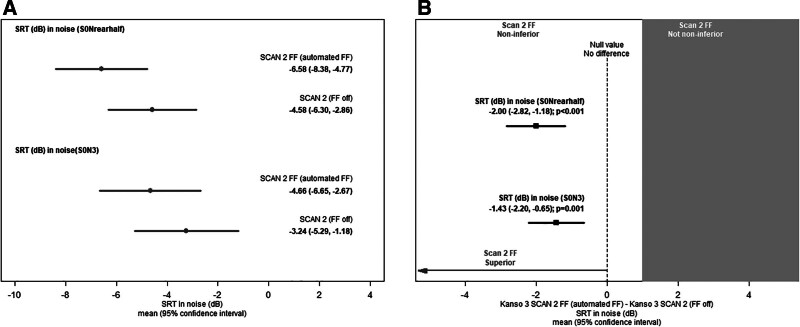
SRT in noise (AuSTIN) with SCAN 2 FF compared with SCAN 2 in the Kanso 3 Sound Processor (n = 17). *A,* Mean SRT sentences in noise score in S0Nrearhalf (top) and S0N3 (bottom) with SCAN 2 FF compared with SCAN 2. *B*, Difference in scores (SCAN 2 FF – SCAN 2) in the S0Nrearhalf (top) and S0N3 (bottom) loudspeaker configurations. Data presented as mean and 95% confidence interval. dB SRT is the minimum intensity level in decibels at which the participants correctly identified 50% words. A lower SRT value corresponds to better speech recognition performance. AuSTIN indicates Australian Sentence Test in Noise; FF, ForwardFocus; SRT, Speech reception threshold.

In the S0Nrearhalf loudspeaker configuration, the mean SRT sentences in noise score for Kanso 3 SCAN 2 FF (automated FF) was −6.58 dB (95% CI, −8.38 dB to −4.77 dB), compared with −4.58 dB (95% CI, −6.30 dB to −2.86 dB; Fig. 4A) with Kanso 3 SCAN 2 (FF off). The difference in means was −2.00 dB (95% CI, −2.82 dB to −1.18 dB; Fig. 4B). Noninferiority of SCAN 2 FF was demonstrated as the upper limit of the 2-sided 95% CI (−1.18 dB) was below the noninferiority margin (1 dB). Testing proceeded to a test of superiority (paired *t* test), which showed that the mean SRT with SCAN 2 FF was statistically significantly lower (better) than that with SCAN 2 (*P* <0.001).

In the S0N3 loudspeaker configuration, the mean SRT sentences in noise scores for Kanso 3 SCAN 2 FF was −4.66 dB (95% CI, −6.65 dB to −2.67 dB), which was lower than the mean score (−3.24 dB) achieved with Kanso 3 SCAN 2 (95% CI, −5.29 dB to −1.18 dB; Fig. 4A). The difference in the means was −1.43 dB (95% CI, −2.20 dB to −0.65 dB; Fig. 4B). Noninferiority of SCAN 2 FF was demonstrated as the upper limit of the 2-sided 95% CI (−0.65 dB) was below the noninferiority margin (1 dB). Testing proceeded to a test of superiority, which showed that the mean SRT with SCAN 2 FF was statistically significantly lower (better) than that with SCAN 2 in S0N3 (*P* = 0.001).

### Speech Recognition in Quiet

To confirm that speech perception in quiet with the Kanso 3 Sound Processor was noninferior to the Kanso 2 Sound Processor, the paired difference in CNC word scores in quiet was compared between these 2 sound processors with FF off. Seventeen paired observations were included in this analysis. The mean CNC words in quiet score for the Kanso 3 Sound Processor was 59.06% (95% CI, 51.91%–66.21%), and 56.53% for the Kanso 2 Sound Processor (95% CI, 49.09–63.97%). The difference between the 2 means was 2.53% (95% CI, −1.41% to 5.47%). Noninferiority was demonstrated as the lower limit of the 2-sided 95% CI for the difference in mean CNC words in quiet scores (−0.41%) was above the noninferiority margin (−10%). However, the improvement of 2.53% in scores was not statistically significant (*P* = 0.087).

### Satisfaction and Acceptance of SCAN 2 FF in the Kanso 3 Sound Processor

At baseline, 15 participants reported using user-controlled FF with a mean self-reported daily use of approximately 1.6 hours. Of these participants, the majority reported being somewhat or very satisfied with the overall sound quality (14/15, 93%) and their overall hearing ability (14/15, 93%) when using FF.

After the second take-home period with the Kanso 3 Sound Processor, 17 participants completed the Kanso 3 Questionnaire to provide their subjective ratings for the 2 programs that were available for use in the Kanso 3 Sound Processor: 1) Program 1 – SCAN 2 FF and 2) Program 2 – SCAN 2 with user-controlled FF. On average, participants reported using Program 1 for 12.6 hours per day while Program 2 was used for 0.6 hours per day, confirming that the participants used the SCAN 2 FF program as advised for prolonged periods during the day.

#### SCAN 2 FF (Program 1) in Kanso 3 Sound Processor

**FIG. 5.**
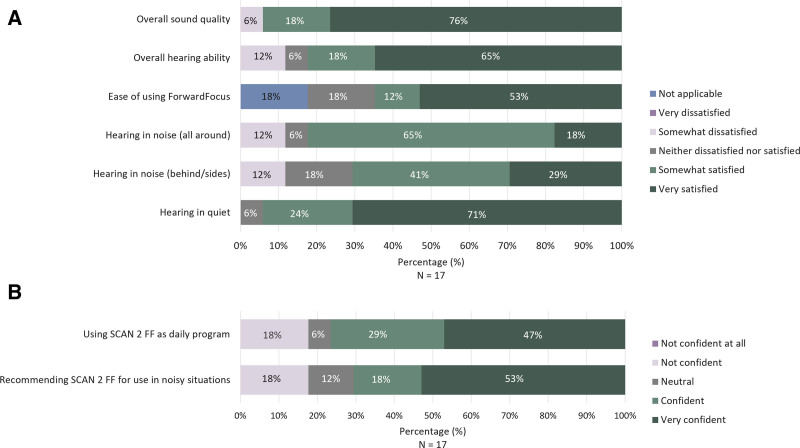
Satisfaction (A) and confidence (B) ratings for SCAN 2 FF in the Kanso 3 Sound Processor. The percentage of participants who indicated each response in the Kanso 3 Questionnaire is shown. Percentages may not total 100% due to rounding. FF, ForwardFocus.

Sixteen (16/17, 94%) participants reported being somewhat or very satisfied with the overall sound quality with SCAN 2 FF, while 14 (14/17, 82%) participants expressed similar satisfaction with their hearing ability when using SCAN 2 FF (Fig. 5A). Eleven (11/17, 65%) participants reported that they were somewhat satisfied or very satisfied with the ease of using SCAN 2 FF. Most participants were somewhat or very satisfied with hearing in noise where the noise sources were all around the participant (14/17, 82%), hearing in noise where noise sources originated from behind and/or sides (12/17, 71%), and hearing in quiet settings (16/17, 94%).

Thirteen (13/17, 79%) participants were confident or very confident in using SCAN 2 FF as their daily program, and 12 (12/17, 72%) were confident or very confident in recommending SCAN 2 FF for use in noisy situations (Fig. 5B).

#### SCAN 2 with user-controlled FF (Program 2) in Kanso 3 Sound Processor

Eight (8/17, 47%) participants reported “not applicable” for all questions related to SCAN 2 with user-controlled FF, as these participants did not use this program at all during the take-home period. Eight (8/17, 47%) participants reported being somewhat or very satisfied with the overall sound quality and overall hearing ability when using SCAN 2 with user-controlled FF.

### Patient-reported Hearing Ability

Eighteen paired observations were used in this analysis. The mean global SSQ12 score for Kanso 3 was 6.07 (95% CI, 5.31–6.83), while the score for participants’ own sound processors was 6.06 (95% CI, 5.29–6.83). The mean difference in scores between the sound processors was 0.10 (95% CI: −0.52 to 0.72). As the lower limit of the 2-sided 95% CI (−0.52) was above −1, noninferiority of the Kanso 3 Sound Processor was established. The mean improvement in SSQ scores (0.10) was not statistically significant (*P* = 0.742).

### Adverse Events

Seven adverse events (AEs) were recorded in this study: 6 participants reported 6 ear-related AEs, while 1 participant reported 1 AE that was not related to the ear. The ear-related AEs were all mild in severity and included the following: tightness at the implant (n = 2)/magnet site (n = 2), pain at the implant site (n = 1), and pain due to stimulation (n = 1). The AE that was not related to the ear was mild (gastroenteritis). All events had resolved at the time of study completion.

## DISCUSSION

The introduction of the SCAN 2 FF program with the Kanso 3 Sound Processor provided significant improvements over SCAN 2 in speech understanding in noisy environments. Speech understanding in quiet conditions remained comparable between the Kanso 3 and Kanso 2 Sound Processor. Following a minimum of 2 weeks of daily use, participants reported high levels of satisfaction and confidence with the SCAN 2 FF program. Additionally, patient-reported hearing ability was consistent between Kanso 3 and their own sound processors.

In the present study, SCAN 2 FF delivered an SRT improvement of 1.43 dB and 2.00 dB compared with SCAN 2 when using the Kanso 3 Sound Processor in the S0N3 and S0Nrearhalf spatially separated speech and noise configurations, respectively, as measured using the AuSTIN test. The benefit observed in the S0N3 condition was comparable to, or slightly greater than, that reported in a previous study evaluating user-controlled FF on the Kanso 2 Sound Processor, which showed a statistically significant 1.2 dB SRT benefit compared with FF off under the same S0N3 condition and speech test (8). SCAN 2 FF was first introduced in the BTE Nucleus 8 Sound Processor, where it demonstrated superior performance over SCAN 2 in both S0N3 and S0Nrearhalf configurations (15). The present study confirms that SCAN 2 FF also shows superior hearing in noise performance compared with SCAN 2 in adults in an OTE configuration. The ability of this feature to dynamically modulate FF strength according to the user’s surroundings may be particularly beneficial for users who cannot or prefer not to manually adjust programs, as it provides a more continuous listening experience and helps ensure that the most optimal settings are used in noisy settings.

Speech perception in quiet remained comparable between the Kanso 3 Sound Processor and its predecessor, the Kanso 2 Sound Processor. This outcome was expected as no major changes were made to the front-end processing strategies used for speech in quiet in the Kanso 3 Sound Processor. In the present study, mean word recognition scores at soft speech levels of 50 dB SPL were 59.06% for the Kanso 3 Sound Processor and 56.53% for the Kanso 2 Sound Processor. These scores align with a previous study on the Kanso 2 Sound Processor, which demonstrated CNC word scores of 62% in 50 dB SPL (8). Together, these data confirm that speech recognition in quiet is maintained in the latest-generation Kanso 3 Sound Processor.

To complement controlled in-booth speech testing, this study collected subjective feedback after at least 2 weeks of take-home use. Most participants reported high satisfaction with overall hearing and hearing in noise using the SCAN 2 FF program, likely due to the convenience of automated FF, which removes the need for manual activation. This aligns with previous findings where 10 out of 15 participants preferred automatic over manual program switching, citing ease of use, less frequent reminders of their hearing impairment, and not needing to worry about selecting the most appropriate program (13). Following the controlled market release of the Kanso 3 Sound Processor System to a small cohort of clinicians (n = 12) for a 2-month, in-clinic evaluation period, survey data indicated that 92% of clinicians reported their patients were satisfied or very satisfied with their upgrade to the overall Kanso 3 Sound Processor system (unpublished). These findings align with prior evidence that OTE sound processors perform well in nonaudiological domains such as comfort, handling, cosmetic appeal, and overall satisfaction (20).

Subjective hearing ability, as measured through the SSQ12, was also similar between the Kanso 3 Sound Processor and participants’ own sound processor. A similar SSQ12 score was expected, given the high level of subjective benefit previously reported with the baseline technology used by participants, which in most cases was the Nucleus 7 Sound Processor (21). The present study’s findings are also consistent with a previous study (22) that found comparable, patient-reported, functional benefits between OTE and BTE sound processors.

This study has several strengths. The take-home period facilitated the assessment of automated FF in real-life listening environments over multiple weeks, rather than solely in controlled settings. To reflect everyday listening scenarios, spatially separated speech and noise configurations were used. The S0N3 configuration aimed to simulate a diffuse noise environment, such as when dining at home or watching television with others, while the S0Nrearhalf configuration aimed to represent a cocktail party or group conversation where the listener is focused on a speaker while dynamic background noise comes from behind (8). One limitation of the study is that the generalizability of the present study’s findings to the broader population of CI users may be limited due to the minimum performance criteria required for enrollment and exclusive use of English-language test materials. Additionally, the sample size was relatively small, which can restrict generalizability; however, the study was adequately powered for its primary endpoint based on a formal sample size calculation. While real-world use was assessed, booth testing was conducted unilaterally. Bilateral CI and bimodal (CI and hearing aid) performance in the booth were not evaluated and may warrant future research to measure everyday listening conditions.

## CONCLUSION

The present study found enhanced speech understanding with the automated SCAN 2 FF program compared with SCAN 2, demonstrating its suitability with the new generation Kanso 3 Sound Processor. The optional automation of FF enables users to benefit from noise reduction without manual program switching and offers users personalization and flexibility to use noise reduction technologies according to their preferences for an optimal listening experience.

## ACKNOWLEDGEMENTS

The authors would like to thank the CI recipients for their participation in this study. We also extend our thanks to Jakson Playford for supporting the interpretation and compilation of study results, and to Ganesha Liyanage and Beejal Vyas-Price for medical writing support. Cochlear, Kanso, Nucleus and Beam are trademarks of Cochlear Limited. Bluetooth and Auracast are trademarks of Bluetooth SIG, Inc.

## FUNDING SOURCES

This work was supported by Cochlear Limited.

## CONFLICTS OF INTERESTS

All authors are current employees of Cochlear Limited, with the exception of MJ, who is no longer affiliated with the company but was employed by Cochlear Limited at the time the study was conducted and this manuscript was drafted.

## DATA AVAILABILITY

The data supporting the findings of this study will not be publicly available. Access to data is restricted to protect proprietary information and allow further analyses of the dataset to support ongoing research.

## Supplementary Material


