# Supplementary material for: Improved Speech Recognition with Automated ForwardFocus in Cochlear Kanso 3 Sound Processor Users
Source: Otol Neurotol Open. 2026 Mar 18;6(1):e087. doi: 10.1097/ONO.0000000000000087 (PMC13012574; doi:10.1097/ONO.0000000000000087)
Supplement: Supplementary file 1 [file ono-6-e087-s001.pdf]

BASELINE QUESTIONNAIRE

Study ID	Subject ID	Date Issued	Date questionnaire completed
CLTD5836			

This questionnaire is designed to help understand more about your experience with your **own sound processor** and related products. To indicate your response, tick a box/circle or write some information in the area provided, according to the type of question. For written answers, please provide as much detail you need to explain your response.

Below is an example of how to complete one of the questions. The question asks: "How satisfied are you with the look and feel of the sound processor?". Tick a circle to indicate your rating.

Example Question: How satisfied are you with the look and feel of the sound processor?

Very dissatisfied	Dissatisfied	Neither dissatisfied nor satisfied	Satisfied	Very Satisfied
○	○	✓	○	○

Please answer all questions. There are no right or wrong answers, so please answer each question as accurately as you can. If you do not understand a question, please answer to the best of your ability.

We ask that you do not make copies of the survey.

All information we receive from you is confidential, and you will not be personally identified when we analyse and report on the results.

The following questions are about your experience with **your own sound processor** and use of the **SCAN program**.

1. How many hours per day do you wear your sound processor(s)?

2. Approximately, how many hours per day do you use your SCAN program? ___________ hours

3. How satisfied are you with your **SCAN Program**? *Select one circle per row.*

**Please rate your satisfaction with the following aspects of your most recent experience with your SCAN program:**	**Very dissatisfied**	**Dissatisfied**	**Neither dissatisfied nor satisfied**	**Satisfied**	**Very Satisfied**	**If you answered, 'Very dissatisfied' or 'Dissatisfied', please briefly tell us why:**
Overall sound quality	○	○	○	○	○	
Overall hearing ability	○	○	○	○	○	
Hearing in noise where the noise sources are from sides and/or back	○	○	○	○	○	
Hearing in noise where the noise sources are all around you	○	○	○	○	○	
Hearing in quiet	○	○	○	○	○	

The following questions are about your experience with **your own sound processor** and use of the **ForwardFocus** noise reduction feature.

4. Do you use ForwardFocus? ○ Yes ○ No

5. **If yes,** approximately, how many hours per day do you use ForwardFocus? ___________ hours

6. **If no,** please tell us why you do not use ForwardFocus *(select all that apply)*:

   ☐ I don't have it

   ☐ It's too difficult to use

   ☐ I forget to turn it on

   ☐ I don't feel that it helps me

   ☐ I don't know about ForwardFocus

   ☐ Other (please specify):____

7. How satisfied are you with **ForwardFocus**?

Select one circle per row. Please only select Not Applicable if you do not use ForwardFocus or if you have not used ForwardFocus in the scenario described.

Please rate your satisfaction with the following aspects of your most recent experience with ForwardFocus:	Very dissatisfied	Dissatisfied	Neither dissatisfied nor satisfied	Satisfied	Very Satisfied	Not Applicable	If you answered, 'Very dissatisfied' or 'Dissatisfied', please briefly tell us why:
Overall sound quality	○	○	○	○	○	○	
Overall hearing ability	○	○	○	○	○	○	

Please rate your satisfaction with the following aspects of your most recent experience with ForwardFocus:	Very dissatisfied	Dissatisfied	Neither dissatisfied nor satisfied	Satisfied	Very Satisfied	Not Applicable	If you answered, 'Very dissatisfied' or 'Dissatisfied', please briefly tell us why:
Hearing in noise where the noise sources are from sides and/or back	○	○	○	○	○	○	
Hearing in noise where the noise sources are all around you	○	○	○	○	○	○	
Hearing in quiet	○	○	○	○	○	○	
Ease of manually turning ForwardFocus ON or OFF	○	○	○	○	○	○	

8. Please rate the usefulness of **ForwardFocus** in different listening environments:

Select one circle per row. Please only select Not Applicable if you do not use ForwardFocus, if you have not used ForwardFocus in the situation described, or if you have not been exposed to the situation.

Situation	Very unhelpful	Unhelpful	Neutral	Helpful	Very helpful	Not applicable	If you answered, 'Very unhelpful or 'Unhelpful', please briefly tell us why:
Café or restaurant	○	○	○	○	○	○	
Meetings at work	○	○	○	○	○	○	
Watching television	○	○	○	○	○	○	

Situation	Very unhelpful	Unhelpful	Neutral	Helpful	Very helpful	Not applicable	If you answered, 'Very unhelpful or 'Unhelpful', please briefly tell us why:
The dinner table at home	○	○	○	○	○	○	
Driving in the car	○	○	○	○	○	○	
Classroom or lectures	○	○	○	○	○	○	
Parties	○	○	○	○	○	○	
Sporting events	○	○	○	○	○	○	
Concerts or live music events	○	○	○	○	○	○	
When using noisy equipment (e.g vacuum cleaner, hairdryer)	○	○	○	○	○	○	
Other (specify) ________________________	○	○	○	○	○		
Other (specify) ________________________	○	○	○	○	○		
Other (specify) ________________________	○	○	○	○	○		

9. Please use the space provided to give us any additional feedback you have about ForwardFocus:

____

The following questions relate to **your hearing experience** in general and **not related to any device or programs**.

10. Thinking of your most recent experience, please tick one circle per row:

Question	Strongly disagree	Disagree	Neutral	Agree	Strongly agree
I feel comfortable being myself.	○	○	○	○	○
I feel anxious when talking to strangers with my hearing device on.	○	○	○	○	○
Wearing my hearing device helps me feel empowered.	○	○	○	○	○
I feel my hearing loss undermines my self-confidence.	○	○	○	○	○

Question	Strongly disagree	Disagree	Neutral	Agree	Strongly agree
I choose when to disclose my hearing loss to strangers.	○	○	○	○	○
I feel self-conscious about wearing my hearing device.	○	○	○	○	○
I try to hide my hearing loss when in public.	○	○	○	○	○
I like the way I look with my hearing device.	○	○	○	○	○
I feel that my hearing loss is an important part of my identity.	○	○	○	○	○
