# Supplementary material for: Improved Speech Recognition with Automated ForwardFocus in Cochlear Kanso 3 Sound Processor Users
Source: Otol Neurotol Open. 2026 Mar 18;6(1):e087. doi: 10.1097/ONO.0000000000000087 (PMC13012574; doi:10.1097/ONO.0000000000000087)
Supplement: Supplementary file 2 [file ono-6-e087-s002.pdf]

**CP1170 Questionnaire**

| Study ID | Subject ID | Date Issued | Date questionnaire completed |
|---|---|---|---|
| CLTD5836 | | | |

This questionnaire is designed to help understand more about your experience with the **research sound processor (Model: CP1170)** and **the listening programs you were provided with**. To indicate your response, tick a box/circle or write some information in the area provided, according to the type of question. For written answers, please provide as much detail you need to explain your response.

Below is an example of how to complete one of the questions. The question asks: "How satisfied are you with the look and feel of the sound processor?". Tick a circle to indicate your rating.

*Example Question:* How satisfied are you with the look and feel of the sound processor?

| Very dissatisfied | Dissatisfied | Neither dissatisfied nor satisfied | Satisfied | Very Satisfied |
|---|---|---|---|---|
| ○ | ○ | ✓ | ○ | ○ |

Please answer all questions. There are no right or wrong answers, so please answer each question as accurately as you can. If you do not understand a question, please answer to the best of your ability. If you are unsure what is meant by the programs provided to you **(Program 1 and Program 2)**, please clarify with the investigator before starting the survey.

We ask that you do not make copies of the survey.

All information we receive from you is confidential, and you will not be personally identified when we analyse and report on the results.

The following questions are about your most recent experience with **research sound processor (CP1170).**

1. How many hours per day do you wear your sound processor(s)? __________ hours

2. Approximately, how many hours per day did you use **Program 1**? __________ hours

3. Approximately, how many hours per day did you use **Program 2**? __________ hours

4. How satisfied are you with the **research sound processor** while using **Program 1?**

*Select one circle per row. Please only select Not Applicable if you do not use ForwardFocus or if you have not used ForwardFocus in the scenario described.*

| Please rate your satisfaction with the following aspects of your most recent experience with Program 1: | Very dissatisfied | Dissatisfied | Neither dissatisfied nor satisfied | Satisfied | Very Satisfied | Not Applicable | If you answered, 'Very dissatisfied' or 'Dissatisfied', please briefly tell us why: |
|---|---|---|---|---|---|---|---|
| Overall sound quality | ○ | ○ | ○ | ○ | ○ | ○ | |
| Overall hearing ability | ○ | ○ | ○ | ○ | ○ | ○ | |
| Hearing in noise where the noise sources are from sides and/or back | ○ | ○ | ○ | ○ | ○ | ○ | |
| Hearing in noise where the noise sources are all around you | ○ | ○ | ○ | ○ | ○ | ○ | |
| Hearing in quiet | ○ | ○ | ○ | ○ | ○ | ○ | |

| Please rate your satisfaction with the following aspects of your most recent experience with Program 1: | Very dissatisfied | Dissatisfied | Neither dissatisfied nor satisfied | Satisfied | Very Satisfied | Not Applicable | If you answered, 'Very dissatisfied' or 'Dissatisfied', please briefly tell us why: |
|---|---|---|---|---|---|---|---|
| Ease of using ForwardFocus | ○ | ○ | ○ | ○ | ○ | ○ | |

5. How satisfied are you with the **research sound processor** while using **Program 2?**

*Select one circle per row. Please only select Not Applicable if you do not use ForwardFocus or if you have not used ForwardFocus in the scenario described.*

| Please rate your satisfaction with the following aspects of your most recent experience with Program 2: | Very dissatisfied | Dissatisfied | Neither dissatisfied nor satisfied | Satisfied | Very Satisfied | Not Applicable | If you answered, 'Very dissatisfied' or 'Dissatisfied', please briefly tell us why: |
|---|---|---|---|---|---|---|---|
| Overall sound quality | ○ | ○ | ○ | ○ | ○ | ○ | |
| Overall hearing ability | ○ | ○ | ○ | ○ | ○ | ○ | |
| Hearing in noise where the noise sources are from sides and/or back | ○ | ○ | ○ | ○ | ○ | ○ | |
| Hearing in noise where the noise sources are all around you | ○ | ○ | ○ | ○ | ○ | ○ | |
| Hearing in quiet | ○ | ○ | ○ | ○ | ○ | ○ | |
| Ease of using ForwardFocus | ○ | ○ | ○ | ○ | ○ | ○ | |

6. Thinking of your most recent experience with the **research sound processor,** please select one option per row:

| How confident would you be with: | Not confident at all | Not confident | Neutral | Confident | Very confident | If you answered 'Very unlikely' or 'Unlikely', please briefly explain why: |
|---|---|---|---|---|---|---|
| Using **Program 1** as your daily program: | ○ | ○ | ○ | ○ | ○ | |
| Using **Program 2** as your daily program: | ○ | ○ | ○ | ○ | ○ | |
| Recommending **Program 1** for use in noisy situations: | ○ | ○ | ○ | ○ | ○ | |

7. Please tell us about the overall ease of use of the **research sound processor.**

*Please select one circle per row.*

| Scenario | Very difficult | Difficult | Neutral | Easy | Very Easy | If you answered 'Very difficult' or 'Difficult', please briefly explain why: |
|---|---|---|---|---|---|---|
| When using Program 1, hearing in noisy sitations is: | ○ | ○ | ○ | ○ | ○ | |
| When I'm not using Program 1, manually turning ForwardFocus ON or OFF is: | ○ | ○ | ○ | ○ | ○ | |
| Adjusting from Program 1 to Program 2 is: | ○ | ○ | ○ | ○ | ○ | |
| Charging the battery is: | ○ | ○ | ○ | ○ | ○ | |

8. Please use the space provided to give us any additional feedback you have about the **research sound processor**:

9. Please rate the usefulness of the **Program 1** in different listening environments.

*Select one circle per row. Please only select Not Applicable if you did not use Program 1, if you have not used Program 1 in the situation described, or if you have not been exposed to the situation.*

| Situation | Very unhelpful | Unhelpful | Neutral | Helpful | Very helpful | Not applicable | If you answered, 'Very unhelpful or 'Unhelpful', please briefly tell us why: |
|---|---|---|---|---|---|---|---|
| Café or restaurant | ○ | ○ | ○ | ○ | ○ | ○ | |
| Meetings at work | ○ | ○ | ○ | ○ | ○ | ○ | |
| Watching television | ○ | ○ | ○ | ○ | ○ | ○ | |
| The dinner table at home | ○ | ○ | ○ | ○ | ○ | ○ | |

| Situation | Very unhelpful | Unhelpful | Neutral | Helpful | Very helpful | Not applicable | If you answered, 'Very unhelpful or 'Unhelpful', please briefly tell us why: |
|---|---|---|---|---|---|---|---|
| Driving in the car | ○ | ○ | ○ | ○ | ○ | ○ | |
| Classroom or lectures | ○ | ○ | ○ | ○ | ○ | ○ | |
| Parties | ○ | ○ | ○ | ○ | ○ | ○ | |
| Sporting events | ○ | ○ | ○ | ○ | ○ | ○ | |
| Concerts or live music events | ○ | ○ | ○ | ○ | ○ | ○ | |
| When using noisy equipment (e.g vacuum cleaner, hairdryer) | ○ | ○ | ○ | ○ | ○ | ○ | |
| *Other (specify)* ___________________________ | ○ | ○ | ○ | ○ | ○ | | |
| *Other (specify)* ___________________________ | ○ | ○ | ○ | ○ | ○ | | |

10. Please rate the usefulness of the **Program 2** in different listening environments.

*Select one circle per row. Please only select Not Applicable if you did not use Program 2, if you have not used Program 2 in the situation described, or if you have not been exposed to the situation.*

| Situation | Very unhelpful | Unhelpful | Neutral | Helpful | Very helpful | Not applicable | If you answered, 'Very unhelpful or 'Unhelpful', please briefly tell us why: |
|---|---|---|---|---|---|---|---|
| Café or restaurant | ○ | ○ | ○ | ○ | ○ | ○ | |
| Meetings at work | ○ | ○ | ○ | ○ | ○ | ○ | |
| Watching television | ○ | ○ | ○ | ○ | ○ | ○ | |
| The dinner table at home | ○ | ○ | ○ | ○ | ○ | ○ | |
| Driving in the car | ○ | ○ | ○ | ○ | ○ | ○ | |
| Classroom or lectures | ○ | ○ | ○ | ○ | ○ | ○ | |
| Parties | ○ | ○ | ○ | ○ | ○ | ○ | |
| Sporting events | ○ | ○ | ○ | ○ | ○ | ○ | |

| Situation | Very unhelpful | Unhelpful | Neutral | Helpful | Very helpful | Not applicable | If you answered, 'Very unhelpful or 'Unhelpful', please briefly tell us why: |
|---|---|---|---|---|---|---|---|
| Concerts or live music events | ○ | ○ | ○ | ○ | ○ | ○ | |
| When using noisy equipment (e.g vacuum cleaner, hairdryer) | ○ | ○ | ○ | ○ | ○ | ○ | |
| *Other (specify)* ___________________________ | ○ | ○ | ○ | ○ | ○ | | |
| *Other (specify)* ___________________________ | ○ | ○ | ○ | ○ | ○ | | |

The following questions relate to **your hearing experience** in general and **not related to any device or programs**.

11. Thinking of your most recent experience, please tick one circle per row:

| Question | Strongly disagree | Disagree | Neutral | Agree | Strongly agree |
|---|---|---|---|---|---|
| I feel comfortable being myself. | ○ | ○ | ○ | ○ | ○ |
| I feel anxious when talking to strangers with my hearing device on. | ○ | ○ | ○ | ○ | ○ |
| Wearing my hearing device helps me feel empowered. | ○ | ○ | ○ | ○ | ○ |

| Question | Strongly disagree | Disagree | Neutral | Agree | Strongly agree |
|---|---|---|---|---|---|
| I feel my hearing loss undermines my self-confidence. | ○ | ○ | ○ | ○ | ○ |
| I choose when to disclose my hearing loss to strangers. | ○ | ○ | ○ | ○ | ○ |
| I feel self-conscious about wearing my hearing device. | ○ | ○ | ○ | ○ | ○ |
| I try to hide my hearing loss when in public. | ○ | ○ | ○ | ○ | ○ |
| I like the way I look with my hearing device. | ○ | ○ | ○ | ○ | ○ |
| I feel that my hearing loss is an important part of my identity. | ○ | ○ | ○ | ○ | ○ |
